# Epley manoeuvre’s efficacy for benign paroxysmal positional vertigo (BPPV) in primary-care and subspecialty settings: a systematic review and meta-analysis

**DOI:** 10.1186/s12875-023-02217-z

**Published:** 2023-12-02

**Authors:** Yusuke Saishoji, Norio Yamamoto, Takashi Fujiwara, Hideki Mori, Shunsuke Taito

**Affiliations:** 1 Department of General Internal Medicine, National Hospital Organization Nagasaki Medical Center, 2- 1001-1 Kubara, Omura, Nagasaki, 856-8562 Japan; 2 Systematic Review Workshop Peer Support Group (SRWS-PSG), Osaka, Japan; 3 Department of Epidemiology, Graduate School of Medicine, Dentistry and Pharmaceutical Sciences, Okayama University, Okayama, 700-8558 Japan; 4 Department of Otolaryngology, Head and Neck Surgery, Kurashiki Central Hospital, 1-1-1 Miwa, Kurashiki, 710-8602 Okayama Japan; 5 Department of Management, Clinical Research Center, Kurashiki Central Hospital, 1-1-1 Miwa, Kurashiki, 710-8602 Okayama Japan; 6 Division of Rehabilitation, Department of Clinical Practice and Support, Hiroshima University Hospital, Kasumi 1-2-3, Minami-ku, Hiroshima, 734-8551 Japan

**Keywords:** Benign paroxysmal positional vertigo, Patient positioning, Primary care, Systematic review, Meta-analysis

## Abstract

**Background:**

Although previous studies have reported general inexperience with the Epley manoeuvre (EM) among general physicians, no report has evaluated the effect of EM on benign paroxysmal positional vertigo (BPPV) in primary care by using point estimates or certainty of evidence. We conducted this systematic review and meta-analysis and clarified the efficacy of EM for BPPV, regardless of primary-care and subspecialty settings.

**Methods:**

Systematic review and meta-analysis of randomised sham-controlled trials of EM for the treatment of posterior canal BPPV in primary-care and subspecialty settings. A primary-care setting was defined as a practice setting by general practitioners, primary-care doctors, or family doctors. A systematic search was conducted in January 2022 across databases, including Cochrane Central Resister of Controlled Trial, MEDLINE, Embase, Cumulative Index of Nursing and Allied Health Literature, World Health Organization International Clinical Trials Registry Platform, and ClinicalTrials.gov. Primary outcomes were the disappearance of subjective symptoms (vertigo), negative findings (Dix–Hallpike test), and all adverse events. We evaluated the certainty of evidence using the Grading of Recommendations, Assessment, Development and Evaluation approach.

**Results:**

Twenty-seven randomised controlled trials were identified. In primary-care settings, EM reduced the subjective symptoms [risk ratio (RR), 3.14; 95% confidence interval (CI), 1.96–5.02]; however, there was no applicable article for all adverse events. In the subspeciality setting, EM reduced the subjective symptoms (RR, 2.42; 95% CI, 1.64–3.56), resulting in an increase in negative findings (RR, 1.81; 95% CI, 1.40–2.34). The evidence exhibited uncertainty about the effect of EM on negative findings in primary-care settings and all adverse events in subspecialty settings.

**Conclusions:**

Regardless of primary-care and subspecialty settings, EM for BPPV was effective. This study has shown the significance of performing EM for BPPV in primary-care settings. EM for BPPV in a primary-care setting may aid in preventing referrals to higher tertiary care facilities and hospitalisation for follow-up.

**Trial registration:**

The study was registered in protocols.io (PROTOCOL INTEGER ID: 51,464) on July 11, 2021.

**Supplementary Information:**

The online version contains supplementary material available at 10.1186/s12875-023-02217-z.

## Background

Benign paroxysmal positional vertigo (BPPV) is a common inner ear disorder. It’s characterized by repeated episodes of vertigo, which are triggered by rapid changes in head position [1]. The most common form of BPPV is the posterior semicircular canals, which account for 85% of cases [2]. However, horizontal canal BPPV is probably much more common than previously recognised [3]. The Dix–Hallpike (DH) manoeuvre is considered the gold standard test for the diagnosis of posterior canal BPPV [4]. Horizontal canal BPPV should be considered when horizontal nystagmus is seen rather than upbeat torsional nystagmus in the DH manoeuvre [3]. There is high-quality and compelling evidence that patients diagnosed with posterior canal BPPV should be offered expeditious treatment with canalith repositioning procedures, commonly referred to as the Epley manoeuvre (EM) [1]. Regarding patients with BPPV, long-term follow-up studies have indicated that vestibular suppressants may not affect symptom resolution; moreover, there is evidence that canalith repositioning procedures are superior to these drugs [5]. EM was first described by Epley in 1992 [6], and systematic review and meta-analysis with small-size randomised controlled trials (RCTs) in the 2014 Cochrane Review [7], including primary-care settings, showed the efficacy of EM for posterior canal BPPV.

Several RCTs on the efficacy of EM on BPPV in primary-care settings [8, 9] have been published since 2014; however, the integrated results of these trials are not yet clear. Furthermore, studies regarding the efficacy of EM in primary-care settings are rare. In the 2014 Cochrane Review [7] of EM for BPPV, 2 of the 11 trials were in primary-care settings [10, 11], and the remainder were conducted in secondary or tertiary care in the otolaryngology departments. It is uncertain whether EM contributes adequately to the treatment of BPPV in the primary-care setting. Therefore, this study aimed to clarify the efficacy of EM for BPPV in primary-care and subspecialty settings.

## Methods

The study was registered in protocols.io (PROTOCOL INTEGER ID: 51,464) [12]. This study was conducted in accordance with the Preferred Reporting Items for Systematic Review and Meta-Analysis (PRISMA) guidelines [13]. We ensured that this study was PRISMA-compliant by consulting the PRISMA 2020 checklist [14] (details provided in Additional file 1).

### Inclusion and exclusion criteria

We included RCTs that assessed the efficacy of EM. Cluster randomised and crossover trials were not included. We did not apply language or country restrictions. We included all articles, including published, unpublished, conference abstracts, and letters. We did not exclude studies based on the observation period or publication year. Participants should have presented with the symptoms of repeated episodes of vertigo, mostly on change of position along with nausea and vomiting. Age, sex, and race did not matter. Participants with a clinical diagnosis of BPPV using the DH test, which proved positional nystagmus reflecting involvement of the posterior canal [4], were included. For posterior canal BPPV, a positive DH test is defined by the presence of upbeating and torsional nystagmus with the top pole of rotation beating toward the affected (downside) ear [4]. The study also included participants with subjective BPPV, where nystagmus is not induced by the DH test; subjective BPPV is an important concept [15]. Participants were diagnosed with BPPV by physicians educated in using EM. EM was administered at the first visit. Repeated manoeuvres or the combination with other interventions that included drugs and rehabilitative exercise were not a concern. Patients who could not tolerate the procedure or had serious heart disease or cervical spine lesions were excluded. The intervention was defined as EM involving a series of four head and body movements from sitting to lying, rolling over, and back to sitting [7]. Control was defined as medication, untreated controls, and sham manoeuvre. A sham manoeuvre consists of laying the patient with the head tilted on the affected side for 5 min. The primary-care setting was defined as a practice setting by general physicians, primary-care physicians, and family physicians.

### Outcomes of interest

The primary outcomes were the disappearance of subjective symptoms (vertigo), negative findings (DH test), and all adverse events. In general, there should be no more than three primary outcomes, including at least one desirable and at least one undesirable outcome [16]. The secondary outcomes were the disappearance of objective symptoms (nystagmus) and Dizziness Handicap Inventory (DHI) score. All outcomes of interest are detailed in Additional file 2.

### Literature search

A systematic search was conducted in January 2022 across databases, including the Central, MEDLINE, Embase, and Cumulative Index of Nursing and Allied Health Literature (details provided in Additional file 3). We also searched the World Health Organization International Clinical Trials Platform Search Portal and ClinicalTrials.gov for ongoing or unpublished trials. We checked the reference lists of studies, including international guidelines [1], reference lists, and articles citing eligible studies. We asked the authors of the original studies for unpublished or Additional data.

### Screening, data extraction, and appraisal

Two independent reviewers (YS, HM) screened titles and abstracts and assessed eligibility based on the full texts. We contacted original authors if relevant data was missing. Disagreements between the two reviewers were resolved by discussion, and if this failed, a third reviewer acted as an arbiter (NY). Two reviewers (YS, HM) performed independent data extraction of the included studies using standardized data collection forms. We used a pre-checked form using 10 randomly selected studies. The form included the information on study design, study population, interventions, and outcomes. Any disagreements were resolved by discussion, and if this failed, a third reviewer acted as an arbiter (NY). Two reviewers (YS, HM) evaluated the risk of bias (ROB) independently using the Risk of Bias 2 [17]. Disagreements between the two reviewers were discussed, and if this failed, a third reviewer (ST) acted as an arbiter, if necessary.

### Data analysis

We pooled the relative risk ratios (RRs) and 95% confidence intervals (CIs) for the following binary variables: disappearance of subjective symptoms (vertigo), negative findings (Dix–Hallpike test), all adverse events, and disappearance of objective symptoms (nystagmus). We pooled the mean differences and 95% CIs for the following continuous variable: Dizziness Handicap Inventory. We summarised adverse events based on the definition in the original article, but we did not perform a meta-analysis. We requested the original authors for the not-presented data.

We performed the intention-to-treat analysis for all dichotomous data. For continuous data, we did not impute missing data based on the recommendation by the Cochrane Handbook [18]. When original studies only reported standard error or a P-value, we calculated the standard deviation based on the method reported by Altman [19]. If these values were unknown when we contacted the authors, the standard deviation was calculated using confidence interval and t-value based on the method indicated in the Cochrane Handbook [18] or validated method [19]. The validity of these methods was analysed using sensitivity analysis.

We evaluated the statistical heterogeneity by visual inspection of the forest plots and calculating the I^2^ statistic (I^2^ values of 0–40%: might not be important; 30–60%: may represent moderate heterogeneity; 50–90%: may represent substantial heterogeneity; and 75–100%: considerable heterogeneity). When there was substantial heterogeneity (I^2^ > 50%), we assessed the reason for the heterogeneity. The Cochrane chi-squared test (Q-test) was performed for I^2^ statistic, and a P-value less than 0.10 was defined as statistically significant.

We searched the clinical trial registry system (ClinicalTrials.gov and International Clinical Trials Platform Search Portal) and performed an extensive literature search for unpublished trials. We assessed the potential publication bias by visual inspection of the funnel plot. The Egger test was also performed; we did not conduct the test when we found fewer than 10 trials or trials with similar sample size.

Meta-analysis was performed using Review Manager software (RevMan 5.4). We used a random-effects model. To elucidate the influence of effect modifiers on results, we evaluated the subgroup analyses of the primary outcomes based on age (≥ 65 years), vertigo severity (above average if using a scale), duration (< 30 days or longer), number of BPPV episodes (first or recurrent episode), number of EM sessions (only once vs. more than once), and EM skills in primary-care settings (whether or not they are educated practitioners) when sufficient data were available. The definition of an educated practitioner is one who has been educated in EM methods by an otolaryngologist or neurologist and observed in practice.

We performed sensitivity analysis for the primary outcomes to assess whether the results of the review were robust to the decisions made during the review process by excluding studies using imputed statistics or excluding studies with high or some concern in the overall assessment of the ROB. We created a summary-of-findings table that included an overall grading of the certainty of the evidence for each primary and secondary outcome, evaluated using the Grading of Recommendations, Assessment, Development and Evaluation (GRADE) approach [20].

## Results

### Study identification

**Fig. 1:**
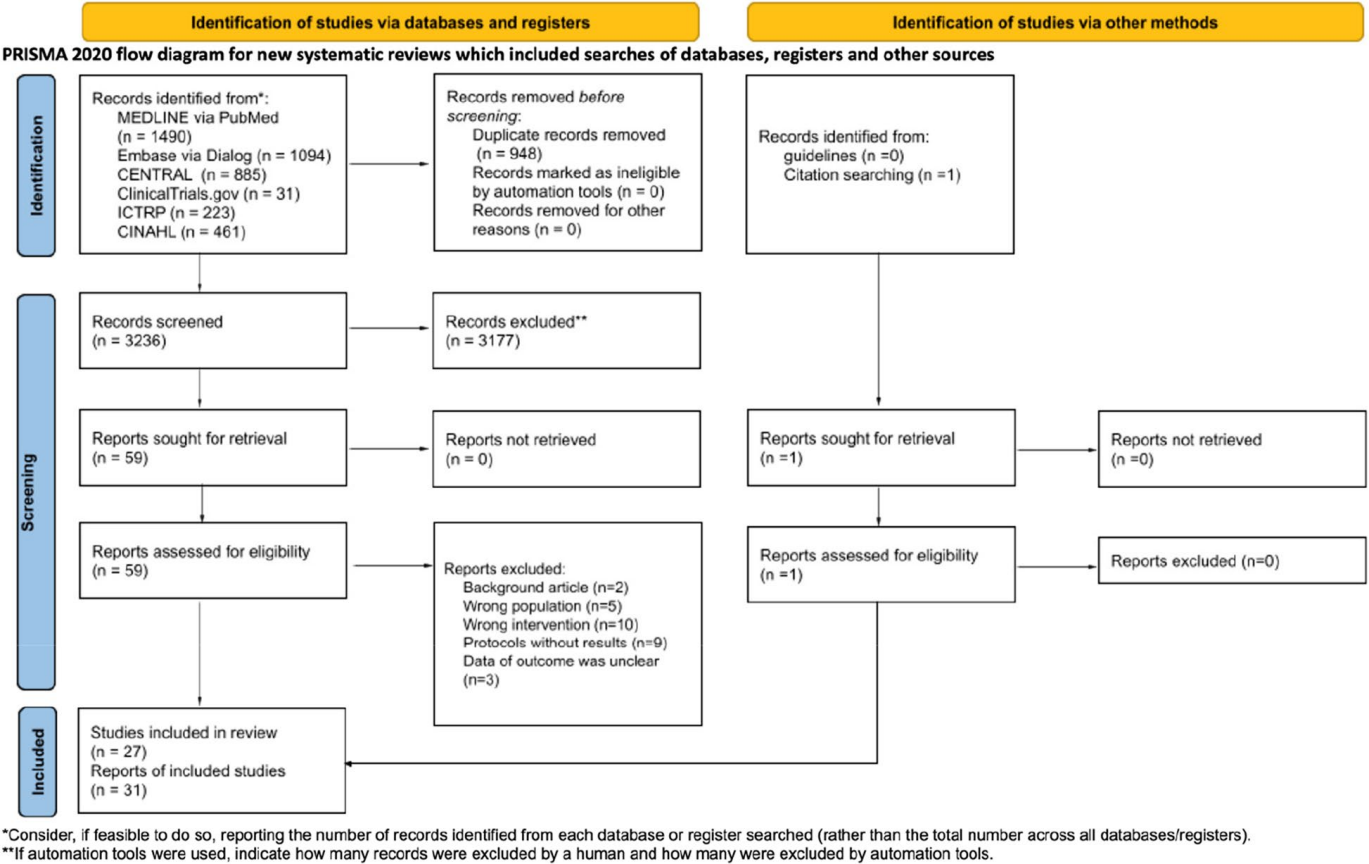
PRISMA 2020 flow diagram

After removing duplicates, we identified 3,236 records during the search conducted in January 2022. We identified 27 RCTs that fulfilled all eligibility criteria and were included in the qualitative synthesis (Fig. 1; details provided in Additional files 4 and 5]). The 27 RCTs provided a pooled sample of 1,629 patients undergoing EM for BPPV. Only 1 RCT [21] did not have valid outcome data.

## Characteristics of the included studies

In total, 4 studies were performed in the primary-care setting [8–11], 15 in the otolaryngology setting [21–35], 4 in the neurology setting [36–39], and 4 in the emergency room setting [40–43]. The duration of the intervention (if measurements were taken at multiple time points, we integrated them using the shortest period) ranged from the first visit to 1 month. We searched for ongoing studies but could not find them. Of the 27 trials analysis, 19 evaluated the outcome of the disappearance of subjective symptoms [8, 10, 11, 22–26, 28, 29, 32, 34, 36–39, 41–43], 16 evaluated the outcome of negative findings [8, 10, 22, 24, 26–28, 30, 31, 33, 35–38, 41, 42], 2 evaluated the outcome of the disappearance of objective symptoms [8, 23], 1 evaluated the outcome of all adverse events [42], 6 evaluated the outcome of DHI-S (the screening version of DHI) [9, 25, 30, 31, 33, 40], and 1 evaluated the outcome of DHI [35]. For three of the studies that evaluated DHI-S [25, 30, 31], we were unable to retrieve outcome data because no reply was received from the authors. In addition, for one study that evaluated DHI [35] results, we were unable to retrieve outcome data. For studies excluded using the full-text screening, bibliographic information was presented in Additional file 6.

### Efficacy of the intervention

Forest plots for each outcome are described in Additional file 7. The summary of findings provides the certainty of the evidence for the outcome in each setting and is listed in Tables 1 and 2.

**Table 1:** Summary of findings: primary-care clinic

Epley manoeuvre compared with the control for BPPV					
Patient or population: Patients with definite diagnosis of BPPV Setting: Primary-care clinic Intervention: Epley manoeuvre Comparison: Sham manoeuvre or no treatment or drug					
Outcomes	**Anticipated absolute effects** ^*^ (95% CI)		Relative effect (95% CI)	№ of participants (studies)	Certainty of the evidence (GRADE)
	**Risk with Control**	**Risk with Epley manoeuvre**			
Disappearance of subjective symptoms (vertigo)	238 events per 1,000 participants	496 events per 1,000 participants (380 to 611)	RR 3.14 (1.96–5.02)	309 (3 RCTs)	⨁⨁◯◯ Low^a,b^
Negative findings (DH test)	406 events per 1,000 participants	592 events per 1,000 participants (292 to 1,000)	RR 1.46 (0.72–2.97)	206 (2 RCTs)	⨁◯◯◯ Very low^a,c^
All adverse events	not pooled	not pooled	-	-	-
Disappearance of objective symptoms (nystagmus)	785 events per 1,000 participants	934 events per 1,000 participants (808 to 1000)	RR 1.19 (1.03–1.38)	127 (1 RCT)	⨁⨁◯◯ Low^c^
Dizziness Handicap Inventory	The mean is 0	MD 2 lower (5.51 lower to 1.51 higher)	-	134 (1 RCT)	⨁⨁◯◯ Low^a,b^
*The risk in the intervention group (and its 95% confidence interval) is based on the assumed risk in the comparison group and the **relative effect** of the intervention (and its 95% CI). **DH test**: Dix–Hallpike test; **BPPV**: benign paroxysmal positional vertigo; **CI**: confidence interval; **MD**: mean difference; **RR**: risk ratio; **SMD**: standardised mean difference;					
**GRADE Working group grades of evidence** **High certainty**: we are very confident that the true effect lies close to that of the estimate of the effect. Moderate certainty: we are moderately confident in the effect estimate: the true effect is likely to be close to the estimate of the effect, but there is a possibility that it is substantially different. Low certainty: our confidence in the effect estimate is limited: the true effect may be substantially different from the estimate of the effect. Very low certainty: we have very little confidence in the effect estimate: the true effect is likely to be substantially different from the estimate of effect.					
Explanations a. Downgraded one level for risk of bias b. Downgraded one level for imprecision c. Downgraded two level for imprecision					

**Table 2:** Summary of findings: otolaryngology or subspecialty settings

Epley manoeuvre compared with the control for BPPV					
Patient or population: Patients with definite diagnosis of BPPV Setting: Otolaryngology or subspecialty settings Intervention: Epley manoeuvre Comparison: Sham manoeuvre or no treatment or drug					
Outcomes	**Anticipated absolute effects** ^*^ (95% CI)		Relative effect (95% CI)	№ of participants (studies)	Certainty of the evidence (GRADE)
	**Risk with Control**	**Risk with Epley manoeuvre**			
Disappearance of subjective symptoms (vertigo)	314 events per 1,000 participants	760 events per 1,000 participants (515 to 1,000)	RR 2.42 (1.64–3.56)	829 (16 RCTs)	⨁⨁◯◯ Low^a,b^
Negative findings (DH test)	443 events per 1,000 participants	802 events per 1,000 participants (621 to 1,000)	RR 1.81 (1.40–2.34)	912 (16 RCTs)	⨁⨁◯◯ Low^a,b^
All adverse events	One study reported all adverse event: 2/24 (Epley manoeuvre group) and 0/26 (control group)		RR 5.40 (0.27–107.09)	50 (1 RCT)	⨁◯◯◯ Very low^a,d^
Disappearance of objective symptoms (nystagmus)	448 events per 1,000 participants	758 events per 1,000 participants (484 to 1,000)	RR 1.69 (1.08–2.66)	58 (1 RCT)	⨁⨁◯◯ Low^d^
Dizziness Handicap Inventory	The mean is 0	MD 8.24 lower (28 lower to 11.51 higher)	-	70 (2 RCTs)	⨁◯◯◯ Very low^a,c,d^
*The risk in the intervention group (and its 95% confidence interval) is based on the assumed risk in the comparison group and the **relative effect** of the intervention (and its 95% CI). **DH test**: Dix–Hallpike test; **BPPV**: benign paroxysmal positional vertigo; **CI**: confidence interval; **MD**: mean difference; **RR**: risk ratio; **SMD**: standardised mean difference;					
**GRADE Working group grades of evidence** **High certainty**: we are very confident that the true effect lies close to that of the estimate of the effect. Moderate certainty: we are moderately confident in the effect estimate: the true effect is likely to be close to the estimate of the effect, but there is a possibility that it is substantially different. Low certainty: our confidence in the effect estimate is limited: the true effect may be substantially different from the estimate of the effect. Very low certainty: we have very little confidence in the effect estimate: the true effect is likely to be substantially different from the estimate of effect.					
Explanations a. Downgraded one level for risk of bias b. Downgraded one level for publication bias c. Downgraded one level for inconsistency d. Downgraded two levels for imprecision					

### Primary outcomes in the primary-care setting

The evidence suggested that EM reduced subjective symptoms (3 studies, 309 participants): RR 3.14; 95% CI 1.96–5.02, I^2^ = 84%; low certainty evidence. The evidence was uncertain about the effect of EM on the negative findings using the DH test (2 studies, 206 participants): RR 1.46; 95% CI 0.72–2.97, I^2^ = 63%; very low certainty evidence.

### Secondary outcomes in the primary-care setting

The disappearance of objective symptoms and DHI-S were measured in one RCT [9]. The evidence suggested that EM reduced objective symptoms slightly (1 study, 127 participants): RR 0.84; 95% CI 0.73–0.97; low certainty evidence. The evidence suggested that EM resulted in little to no difference in DHI-S (1 study, 134 participants): mean difference − 2; 95% CI -5.51 to 1.51; low certainty evidence.

### Primary outcomes in the otolaryngology or subspecialty settings

The evidence suggested that EM reduced subjective symptoms (16 studies, 829 participants): RR 2.42; 95% CI 1.64–3.56, I^2^ = 84%; low certainty evidence. The evidence suggested that EM resulted in an increase in negative findings using the DH test (16 studies, 912 participants): RR 1.81; 95% CI 1.40–2.34, I^2^ = 79%; low certainty evidence. The evidence was uncertain about the effect of EM on all adverse events: one study [42] (50 participants) reported all adverse events.

### Secondary outcomes in the otolaryngology or subspecialty settings

The disappearance of objective symptoms was measured in one RCT [23], and DHI-S was measured in two RCTs [33, 40]. The evidence suggested that EM reduced objective symptoms slightly (1 study, 58 participants): RR 1.69; 95% CI 1.08–2.66; low certainty evidence. The evidence was uncertain about the effect of EM on DHI-S (2 studies, 70 participants): mean difference − 8.24; 95% CI -28 to 11.51; very low certainty evidence.

### Quality assessment

Risk of Bias 2 was used for the evaluation of the ROB. Most studies were at high or some concern ROB, as per the Cochrane ROB assessment tool (details provided in Additional file 8). In the primary-care and otolaryngology or subspecialty settings, the disappearance of subjective symptoms was a subjective assessment and resulted in a high ROB. Two studies of negative findings in the primary-care setting [8, 10] had some concern ROB because one study demonstrated a low ROB except for the randomisation process, and the other demonstrated a low ROB except for deviations from the intended intervention. There was no study about all adverse events in primary-care settings.

Two studies of negative findings in the otolaryngology or subspecialty settings [24, 36] had a low ROB, and six studies [8, 10, 35, 37, 41, 42] had some concern ROB. The other studies demonstrated a high ROB. One study [42] of all adverse events in the otolaryngology or subspecialty settings had a high ROB because the outcome was measured. There was evidence of publication bias using Egger’s test (P < 0.001) in a reduction of subjective symptoms and an increase in negative findings using the DH test in the otolaryngology or subspecialty settings.

### Subgroup analysis and sensitivity analysis

The prespecified subgroup analyses for the primary outcomes revealed no significant differences among subgroups (details provided in Additional file 9). We were unable to perform subgroup analyses for items ‘vertigo severity’, ‘number of BPPV episodes (first or recurrent episode)’, and number of EM sessions (only once vs more than once)’ because there were no applicable studies.

The prespecified sensitivity analysis for the primary outcomes was carried out because there was no study that used imputed statistics, but two studies [24, 36] were a low ROB in the outcome of negative findings using the DH test in the otolaryngology or subspecialty settings. Similar results were obtained in the sensitivity analysis (details provided in Additional file 10).

## Discussion

This study suggests that regardless of primary-care and subspecialty settings, EM for BPPV was effective. The primary-care setting has fewer studies and a smaller sample size than that in the subspecialty setting and showed very low to low evidence for improvement in the subjective and objective endpoints. In the primary-care setting, it has been pointed out that EM is not adequately performed due to the level of skill and lack of confidence using the DH test [44]. EM is often not performed, and the patient is treated with oral medications [45]. However, EM can be learned through video-based training [46] and can be performed within a 10-minute consultation [44], making it valuable for the primary-care setting. Not only is the treatment of BPPV with EM effective, being able to address the problem definitively in the office is satisfying for both the patient and doctor.

Previous studies have not presented point estimates and CoE separately for primary-care and other settings. This study assessed the efficacy of EM for BPPV in the primary-care setting. Although not specific to the primary-care setting, the Cochrane Review examining the efficacy of EM for BPPV included two trials of the primary-care setting [7] and showed that complete resolution of vertigo occurred significantly more often in the EM group compared with the sham manoeuvre or control group, and conversion from a positive to a negative DH test significantly favoured the EM group compared with the sham manoeuvre or control group. The efficacy of EM was demonstrated in the primary-care setting and Cochrane Review.

Furthermore, subgroup analyses of age and duration were performed to search for causes of heterogeneity, but no significant differences were found. Studies evaluated in all settings have reported no significant difference in efficacy between older and younger patients. However, many older patients with BPPV may have difficulty performing canalith repositioning procedures due to various orthopaedic and vascular problems, such as limited range of motion of the cervical spine, kyphosis, or a history of vertebrobasilar insufficiency or stroke, and thus require careful enforcement [47]. Regarding the symptom duration, a BPPV vertigo attack lasts approximately 30 s; there is no obvious reason why EM should be more or less effective at different times between onset and spontaneous resolution if the mechanism of onset is similar between cases [7]. This study showed no significant differences in the subgroup analysis of the symptom duration persistence divided by 30 days, consistent with this hypothesis.

This study has several strengths. First, we registered the protocol according to the PRISMA guidelines and employed a robust methodology with comprehensive evidence searching. Second, we used the GRADE approach for assessing the certainty of evidence (CoE) and referred to the Cochrane Handbook [20]. Third, this is the first study to report point estimation and CoE separately for primary-care and subspecialty settings. The 2014 Cochrane Review [7] included a section for EM in the primary-care setting but not for CoE. In addition, we were able to add and analyse the literature on the primary-care setting since 2014, when the Cochrane Review was published.

This study has several potential limitations. First, long-term effects could not be evaluated because the shortest timing was used as the timing for evaluating outcomes. BPPV is a spontaneously resolving disease with an average symptom duration of 39 days [48]. Therefore, the follow-up duration in the included studies was enough for clinical assessment. Second, we were unable to perform subgroup analysis for dizziness severity, presence or absence of recurrence, and repeated EM. Third, experienced physicians performed EM for BPPV in the included studies. A previous study reported the general lack of experience using the DH test among general physicians [8]. As the procedure is simple, the effect size may differ depending on the therapist’s experience. This study evaluated the efficacy of the intervention based on the assumption that EM skills were mastered. Recent related articles have shown that even non-specialists can achieve excellent results if they are trained in the technique [49, 50]. Fourth, we did not perform a sensitivity analysis of studies wherein DH was performed as an objective measure in follow-up; consequently, we may not have been able to assess the true efficacy of the intervention.

This study demonstrated the efficacy of EM for BPPV in the primary-care setting, but it was based on several small-scale studies with a high ROB. We believe that more large-scale, high-quality studies are needed to estimate more accurate efficacy. We were unable to perform subgroup analysis for dizziness severity, presence or absence of recurrence, and repeated EM. Future studies need to assess whether it could be a source of heterogeneity.

## Conclusions

Regardless of primary-care and subspecialty settings, EM for BPPV was effective. The results of this study support EM for BPPV in the primary-care setting. EM for BPPV in primary-care settings may aid in preventing referrals to higher tertiary-care facilities and hospitalisation for follow-up. Furthermore, results reported herein are expected to provide further insight into the cost-effectiveness of implementing EM in the primary care setting.

### Supplementary Information

**Additional file 1.** PRISMA 2020 Checklist.

**Additional file 2.** Outcomes of interest.

**Additional file 3.** Search strategies.

**Additional file 4.** Characteristics of the included Studies (primary-care setting) (N = 4).

**Additional file 5.** Characteristics of the included studies (otolaryngology or subspecialty settings) (N = 23).

**Additional file 6.** Characteristics of studies excluded from qualitative and quantitative synthesis.

**Additional file 7.** Forest plots for each outcome.

**Additional file 8.** Risk of bias table.

**Additional file 9.** Subgroup analysis.

**Additional file 10.** Sensitivity analysis.

## Data Availability

The datasets generated or analysed during this study are included in this published article and its supplementary information files.

## Abbreviations

BPPV: Benign Paroxysmal Positional Vertigo
EM: Epley Manoeuvre
RR: Risk Ratio
CI: Confidence Interval
DH: Dix–Hallpike
RCTs: Randomised Controlled Trials
PRISMA: Preferred Reporting Items for Systematic Review and Meta-Analysis
DHI: Dizziness Handicap Inventory
ROB: Risk of Bias
GRADE: Grading of Recommendations, Assessment, Development and Evaluation
DHI-S: Screening version of DHI
CoE: Certainty of Evidence
