# Supplementary material for: Epley manoeuvre’s efficacy for benign paroxysmal positional vertigo (BPPV) in primary-care and subspecialty settings: a systematic review and meta-analysis
Source: BMC Prim Care. 2023 Dec 2;24:262. doi: 10.1186/s12875-023-02217-z (PMC10693044; doi:10.1186/s12875-023-02217-z)
Supplement: Supplementary file 1 — Additional file 1. PRISMA 2020 Checklist. [file 12875_2023_2217_MOESM1_ESM.docx]

| Additional file 4. Characteristics of the included studies (primary-care setting) (N = 4) | | | | |  | |  | |
| --- | --- | --- | --- | --- | --- | --- | --- | --- |
| Study Author, Year | Condition/Symptoms, Setting | Comparison | No. Randomised | Outcomes Assessed Here  (Outcome Assessment Tool) | | Time of Outcome Assessment | |  |
| Munoz, 2007 | Positional vertigo and Positive DH test, Primary care | Epley vs Sham manoeuvre | 81 | Disappearance of subjective symptoms (vertigo),  Negative findings (DH test) | | First visit | |  |
| Xie, 2012 | A typical history of BPPV and positive DH test, Primary care | Epley plus postual restrictions vs Postual restrictions | 103 | Disappearance of subjective symptoms (vertigo) | | 4 days | |  |
| Moreno, 2019 | A typical history of BPPV and positive DH test, Primary care | Epley vs Sham manoeuvre | 134 | Disappearance of subjective symptoms (vertigo),  Negative findings (DH test),  Disappearance of objective symptoms (nystagmus) | | 1 week | |  |
| Carrillo Munos, 2021 | A typical history of BPPV and Clinically suspected to have posterior canal BPPV, Primary care | Epley plus Drug vs Drug | 134 | Dizziness Handicap Inventory | | 1 week | |  |

DH test, Dix–Hallpike test; BPPV, benign paroxysmal positional vertigo
