# Supplementary material for: Epley manoeuvre’s efficacy for benign paroxysmal positional vertigo (BPPV) in primary-care and subspecialty settings: a systematic review and meta-analysis
Source: BMC Prim Care. 2023 Dec 2;24:262. doi: 10.1186/s12875-023-02217-z (PMC10693044; doi:10.1186/s12875-023-02217-z)
Supplement: Supplementary file 2 — Additional file 2. Outcomes of interest. [file 12875_2023_2217_MOESM2_ESM.docx]

| Additional file 6. Characteristics of studies excluded from qualitative and quantitative synthesis | |
| --- | --- |
|  |  |
| Study (researcher, publication data, or trial registry number) | Reason for exclusion |
| Aso S, et al. Equilibrium Research. 1995;54:547-52. | Background article |
| Kerber KA. Ann N Y Acad Sci. 2015;1343:106-12. | Background article |
| Itaya T, et al. ORL J Otorhinolaryngol Relat Spec. 1997;59:155-8. | Wrong population |
| Richard W, et al. Ear Nose Throat J. 2005;84:22-5. | Wrong population |
| Ruckenstein MJ. Laryngoscope. 2001;111:940-5. | Wrong population |
| Seo T, et al. Otol Neurotol. 2007;28:917-9. | Wrong population |
| Waleem SS, et al. J Ayub Med Coll Abbottabad. 2008;20:77-9. | Wrong population |
| Cohen HS, et al. Otolaryngology - Head and Neck Surgery. 2004;131:107. | Wrong intervention |
| Firrisi L, et al. Clinical Otolaryngology and Allied Sciences. 2004;29:412. | Wrong intervention |
| Herdman SJ, et al. Arch Otolaryngol Head Neck Surg. 1993;119:450-4. | Wrong intervention |
| Kim M-B, et al. Laryngoscope. 2014;124(10):2400-3. | Wrong intervention |
| Mossman S, et al. Journal of the Neurological Sciences. 2001;187:S233. | Wrong intervention |
| Niamatullah NY. Pakistan Journal of Otolaryngology. 2004;20:3-5. | Wrong intervention |
| Strickland C, Russell R. Journal of Family Practice. 2003;52(12):971-3. | Wrong intervention |
| Uematu M, et al. Acta Otolaryngol Suppl. 1991;481:624-5. | Wrong intervention |
| Vital V, et al. B-ENT. 2010;6:9-13. | Wrong intervention |
| y WUW, et al. Modern medicine & health. 2011;13:005. | Wrong intervention |
| García-Muñoz C, et al. BMJ Open. 2021;11:1. | Protocols without results |
| García-Muñoz C, et al. BMJ Open. 2021;11(4). | Protocols without results |
| Ballve Moreno JL, et al. Trials. 2014;15:179. | Protocols without results |
| Karimi-Sari H. IRCT2016101717413N19 | Protocols without results |
| Oh S. NCT01822002 | Wrong intervention |
| Imai T. UMIN000016421 | Wrong intervention |
| Munoz J. NCT00182273 | Protocols without results |
| Cohen H. NCT00000359 | Protocols without results |
| García-Muñoz C. NCT04578262 | Protocols without results |
| García-Muñoz C, et al. BMJ Open. 2021;11:e046510. | Data of outcome was unclear |
| Palaniappan R. ISRCTN84087880 | Data of outcome was unclear |
| Park HJ. NCT02029508 | Data of outcome was unclear |
