# Supplementary material for: Epley manoeuvre’s efficacy for benign paroxysmal positional vertigo (BPPV) in primary-care and subspecialty settings: a systematic review and meta-analysis
Source: BMC Prim Care. 2023 Dec 2;24:262. doi: 10.1186/s12875-023-02217-z (PMC10693044; doi:10.1186/s12875-023-02217-z)
Supplement: Supplementary file 3 — Additional file 3. Search strategies. [file 12875_2023_2217_MOESM3_ESM.docx]

Additional file 3. Search strategies

| Central search strategy |
| --- |
| #1 [mh Vertigo] OR [mh Dizziness] OR Vertigo:ti,ab OR Dizziness:ti,ab OR BPPV:ti,ab OR "Familial Vestibulopathy":ti,ab  #2 [mh "Patient Positioning"] OR [mh "Physical Therapy Modalities"] OR [mh Posture] OR [mh "Exercise Movement Techniques"] OR [mh "Head Movements"] OR "multidisciplinary treatment":ti,ab OR "Patient Positioning":ti,ab OR ("physical therapy" NEAR/2 modalit*):ti,ab OR "Combined Modality Therapy":ti,ab OR Posture:ti,ab OR ("exercise movement" NEAR/2 technique*):ti,ab OR ("head" NEAR/2 movement*):ti,ab OR "canalith repositioning":ti,ab OR epley*:ti,ab OR semont*:ti,ab OR canalith*:ti,ab OR otolith*:ti,ab OR particle:ti,ab OR position*:ti,ab OR reposition*:ti,ab OR maneuver*:ti,ab OR manoeuvr*:ti,ab  #3  #1 AND #2 |

| MEDLINE (via PubMed) search strategy |
| --- |
| #1 Vertigo[mh]  #2 Dizziness[mh]  #3 vertigo[tiab]  #4 dizziness[tiab]  #5 BPPV[tiab]  #6 "familial vestibulopathy"[tiab]  #7 #1 OR #2 OR #3 OR #4 OR #5 OR #6  #8 Patient Positioning[mh]  #9 Physical Therapy Modalities[mh]  #10 Posture[mh]  #11 Exercise Movement Techniques[mh]  #12 Head Movements[mh]  #13 "multidisciplinary treatment"[tiab]  #14 "Physical Therapy Modalit*"[tiab]  #15 Posture[tiab]  #16 "Exercise Movement Technique*"[tiab]  #17 "Head Movement*"[tiab]  #18 epley*[tiab]  #19 semont*[tiab]  #20 canalith*[tiab]  #21 otolith*[tiab]  #22 particle[tiab]  #23 position*[tiab]  #24 reposition*[tiab]  #25 maneuver*[tiab]  #26 manoeuvr*[tiab]  #27 #8 OR #9 OR #10 OR #11 OR #12 OR #13 OR #14 OR #15 OR #16 OR #17 OR #18 OR #19 OR #20 OR #21 OR #22 OR #23 OR #24 OR #25 OR #26  #28 #7 AND #27  #29 randomized controlled trial [pt]  #30 controlled clinical trial [pt]  #31 randomized [tiab]  #32 placebo [tiab]  #33 drug therapy [sh]  #34 randomly [tiab]  #35 trial [tiab]  #36 groups [tiab]  #37 #29 OR #30 OR #31 OR #32 OR #33 OR #34 OR #35 OR #36  #38 animals [mh] NOT humans [mh]  #39 #37 NOT #38  #40 #28 AND #39 |

| Embase (via ProQuest Dialog) search strategy |
| --- |
| S1 (EMB.EXACT.EXPLODE("vertigo"))  S2 (EMB.EXACT.EXPLODE("dizziness"))  S3 (ab(vertigo) OR ti(vertigo))  S4 (ab(dizziness) OR ti(dizziness))  S5 (ab(BPPV) OR ti(BPPV))  S6 (ab(“familial vestibulopathy”) OR ti(“familial vestibulopathy”))  S7 S1 OR S2 OR S3 OR S4 OR S5 OR S6  S8 (EMB.EXACT.EXPLODE("patient positioning"))  S9 (EMB.EXACT.EXPLODE("physiotherapy"))  S10 (EMB.EXACT.EXPLODE("body position"))  S11 (EMB.EXACT.EXPLODE("kinesiotherapy"))  S12 (EMB.EXACT.EXPLODE("head movement"))  S13 (ab(“multidisciplinary treatment”) OR ti(“multidisciplinary treatment”))  S14 (ab(Physical Therapy Modalit*) OR ti(Physical Therapy Modalit*))  S15 (ab(Posture) OR ti(Posture))  S16 (ab(Exercise Movement Technique*) OR ti(Exercise Movement Technique*))  S17 (ab(Head Movement*) OR ti(Head Movement*))  S18 (ab(epley*) OR ti(epley*))  S19 (ab(semont*) OR ti(semont*))  S20 (ab(canalith*) OR ti(canalith*))  S21 (ab(otolith*) OR ti(otolith*))  S22 (ab(particle) OR ti(particle))  S23 (ab(position*) OR ti(position*))  S24 (ab(reposition*) OR ti(reposition*))  S25 (ab(maneuver*) OR ti(maneuver*))  S26 (ab(manoeuvr*) OR ti(manoeuvr*))  S27 S8 OR S9 OR S10 OR S11 OR S12 OR S13 OR S14 OR S15 OR S16 OR S17 OR S18 OR S19 OR S20 OR S21 OR S22 OR S23 OR S24 OR S25 OR S26  S28 S7 AND S27  S29 (ab(random*) OR ti(random*)) OR (ab(clinical NEAR/1 trial*) OR ti(clinical NEAR/1 trial*)) OR (EMB.EXACT("health care quality"))  S30 S28 AND S29 |

| Cumulative Index of Nursing and Allied Health Literature search strategy |
| --- |
| S1 ((MH "vertigo+") OR (MH "dizziness+") OR (TI vertigo OR AB vertigo) OR (TI dizziness OR AB dizziness) OR (TI BPPV OR AB BPPV) OR (TI "familial vestibulopathy" OR AB "familial vestibulopathy"))  S2 ((MH "patient positioning+") OR (MH "physical therapy modalities+") OR (MH "Posture+") OR (MH "exercise movement techniques+") OR (MH "head movements+") OR (TI "multidisciplinary treatment" OR AB "multidisciplinary treatment") OR (TI "physical therapy modalit*" OR AB "physical therapy modalit*") OR (TI Posture OR AB Posture) OR (TI "exercise movement technique*" OR AB "exercise movement technique*") OR (TI "head movement*" OR AB "head movement*") OR (TI epley* OR AB epley*) OR (TI semont* OR AB semont*) OR (TI canalith* OR AB canalith*) OR (TI otolith* OR AB otolith*) OR (TI particle OR AB particle) OR (TI position* OR AB position*) OR (TI reposition* OR AB reposition*) OR (TI maneuver* OR AB maneuver*) OR (TI manoeuvr* OR AB manoeuvr*))  S3 S1 AND S2  S4 (((MH randomized controlled trials) OR (MH double-blind studies) OR (MH single-blind studies) OR (MH random assignment) OR (MH pretest-posttest design) OR (MH cluster sample) OR (TI (randomised OR randomized)) OR( AB (random*)) OR (TI (trial)) OR (MH (sample size) AND AB (assigned OR allocated OR control)) OR (MH (placebos)) OR( PT (randomized controlled trial)) OR (AB (control W5 group)) OR (MH (crossover design) OR MH (comparative studies))))NOT ((((MH animals+) OR( MH (animal studies)) OR (TI (animal model*))) NOT (MH (human))))  S5 S3 AND S4 |

| International Clinical Trials Platform Search Portal search strategy |
| --- |
| Condition:  Vertigo OR Dizziness OR "Familial Vestibulopathy" OR BPPV  Intervention:  "physical therapy modalities" OR Posture OR "Exercise Movement Techniques" OR "head movement*" OR epley* OR particle OR maneuver* OR manoeuvr* OR "multidisciplinary treatment" |

| ClinicalTrials.gov search strategy |
| --- |
| Condition or disease  Vertigo OR Dizziness OR "Familial Vestibulopathy" OR BPPV  Intervention  "physical therapy modalities" OR Posture OR "Exercise Movement Techniques" OR "head movement*" OR epley* OR particle OR maneuver* OR manoeuvr* OR [] "multidisciplinary treatment" |
