# Supplementary material for: Epley manoeuvre’s efficacy for benign paroxysmal positional vertigo (BPPV) in primary-care and subspecialty settings: a systematic review and meta-analysis
Source: BMC Prim Care. 2023 Dec 2;24:262. doi: 10.1186/s12875-023-02217-z (PMC10693044; doi:10.1186/s12875-023-02217-z)
Supplement: Supplementary file 4 — Additional file 4. Characteristics of the included Studies (primary-care setting) (N = 4). [file 12875_2023_2217_MOESM4_ESM.docx]

| Additional file 2. Outcomes of interest | |  |  |
| --- | --- | --- | --- |
|  |  |  |  |
|  | Outcome | Definition | Timing of measurement |
| Primary outcome | Disappearance of subjective symptoms (vertigo) | A state in which the symptoms have disappeared | Immediately after the treatment, 1 week, 2 weeks, 1 month, and 1 year later (If measurements are taken at multiple time points, we integrated them using the shortest period.) |
|  | Negative findings (DH test) | A doctor describes a state in which the symptoms have completely disappeared [In the DH test, signs of dizziness and nystagmus (involuntary eye movement) are considered positive (1).] | Immediately after the treatment, 1 week, 2 weeks, 1 month, and 1 year later (If measurements are taken at multiple time points, we integrated them using the shortest period.) |
|  | All adverse events | Adverse events that are set by the original authors | Throughout the entire study period |
| Secondary outcome | Disappearance of objective symptoms (nystagmus) | A doctor describes a state in which the symptoms have completely disappeared. | Immediately after the treatment, 1 week, 2 weeks, 1 month, and 1 year later (If measurements are taken at multiple time points, we integrated them using the shortest period.) |
|  | Dizziness Handicap Inventory (DHI) score | The 25-item Dizziness Handicap Inventory is developed to evaluate the self-perceived handicapping effects imposed by vestibular system disease. [The items are subgrouped into three content domains representing functional, emotional, and physical aspects of dizziness and unsteadiness (2).] | Immediately after the treatment, 1 week, 2 weeks later, 1 month, and 1 year later (If measurements are taken at multiple time points, we integrated them using the shortest period.) |

DH test, Dix–Hallpike test

1. David J. Magee PhD B, CM and Robert C. Manske PT, DPT, MEd, SCS, ATC, CSCS. Orthopedic Physical Assessment. Head and Face. 7 ed: Elsevier; 2021. p. 73-163.e4.

2. Jacobson GP, Newman CW. The development of the Dizziness Handicap Inventory. Arch Otolaryngol Head Neck Surg. 1990;116:424-7.
