# Supplementary material for: Epley manoeuvre’s efficacy for benign paroxysmal positional vertigo (BPPV) in primary-care and subspecialty settings: a systematic review and meta-analysis
Source: BMC Prim Care. 2023 Dec 2;24:262. doi: 10.1186/s12875-023-02217-z (PMC10693044; doi:10.1186/s12875-023-02217-z)
Supplement: Supplementary file 5 — Additional file 5. Characteristics of the included studies (otolaryngology or subspecialty settings) (N = 23). [file 12875_2023_2217_MOESM5_ESM.docx]

Additional file 9. Subgroup analysis

1. Older adult participants aged ≥ 65 years

Disappearance of subjective symptoms (vertigo)


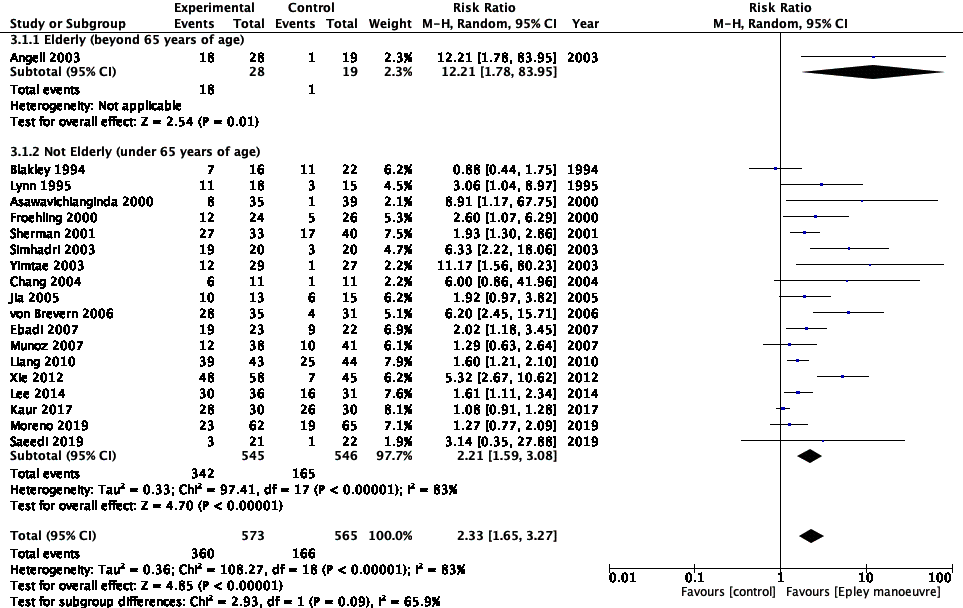


 Negative findings (DH test)

  Unavailable

 All adverse event

  Unavailable

DH test, Dix–Hallpike test; CI, confidence interval

2. Vertigo severity (above average if using a scale; for the participants)

Disappearance of subjective symptoms (vertigo)

Unavailable

Negative findings (DH test)

  Unavailable

All adverse event

Unavailable

DH test, Dix–Hallpike test;

3. Duration (< 30 days or longer; for the participants)

Disappearance of subjective symptoms (vertigo)

Unavailable

Negative findings (DH test)


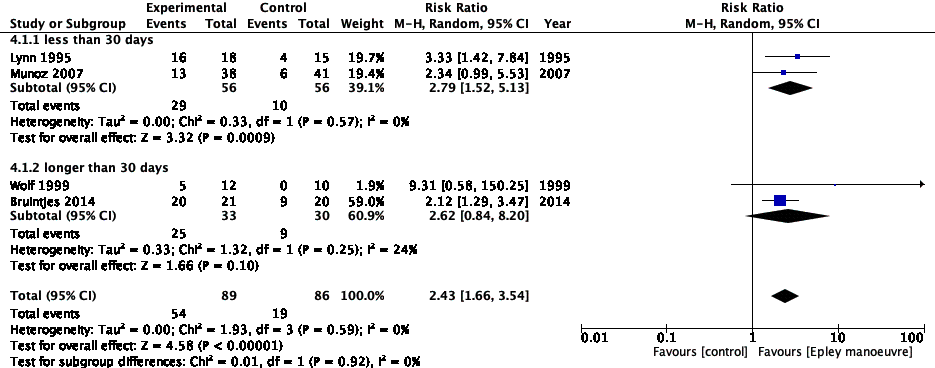


 All adverse event

  Unavailable

DH test, Dix–Hallpike test; CI, confidence interval

4. First episode or recurrent BPPV (for the participants)

Disappearance of subjective symptoms (vertigo)

Unavailable

Negative findings (DH test)

Unavailable

All adverse event

Unavailable

BPPV, benign paroxysmal positional vertigo; DH test, Dix–Hallpike test

5. Group that performed EM only once vs group that performed EM more than once (for the intervention)

Disappearance of subjective symptoms (vertigo)

Unavailable

Negative findings (DH test)

Unavailable

All adverse event

Unavailable

DH test, Dix–Hallpike test; EM, Epley manoeuvre

6. Group that performed EM by educated practitioners vs group that performed EM by uneducated practitioners or unknown in primary care settings

Disappearance of subjective symptoms (vertigo)


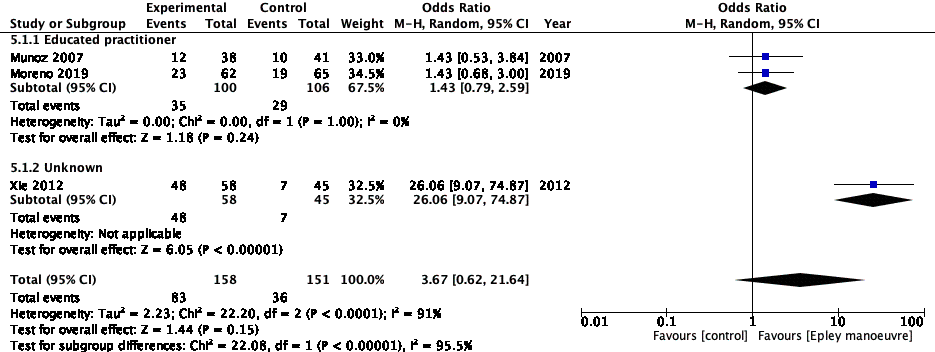


Negative findings (DH test)

  Unavailable

All adverse event

Unavailable

EM, Epley manoeuvre
