# Supplementary figures and images for: Epley manoeuvre’s efficacy for benign paroxysmal positional vertigo (BPPV) in primary-care and subspecialty settings: a systematic review and meta-analysis
Source: BMC Prim Care. 2023 Dec 2;24:262. doi: 10.1186/s12875-023-02217-z (PMC10693044; doi:10.1186/s12875-023-02217-z)

Additional file 10. Sensitivity analysis


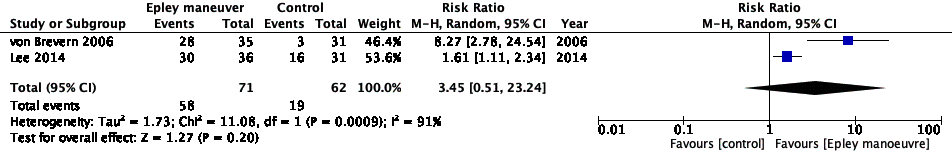


CI, confidence interval

Supplement: Supplementary file 7 — Additional file 7. Forest plots for each outcome. [file 12875_2023_2217_MOESM7_ESM.docx]
