# Supplementary material for: Epley manoeuvre’s efficacy for benign paroxysmal positional vertigo (BPPV) in primary-care and subspecialty settings: a systematic review and meta-analysis
Source: BMC Prim Care. 2023 Dec 2;24:262. doi: 10.1186/s12875-023-02217-z (PMC10693044; doi:10.1186/s12875-023-02217-z)
Supplement: Supplementary file 8 — Additional file 8. Risk of bias table. [file 12875_2023_2217_MOESM8_ESM.docx]

Additional file 7. Forest plots for each outcome

**Patients with BPPV present at primary care clinics.**

Primary outcomes

1. Disappearance of subjective symptoms (vertigo)

**
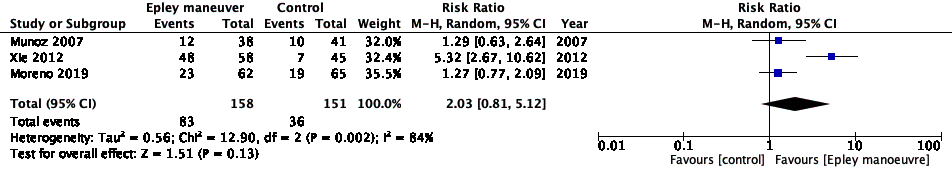
**

2. Negative findings (DH test)


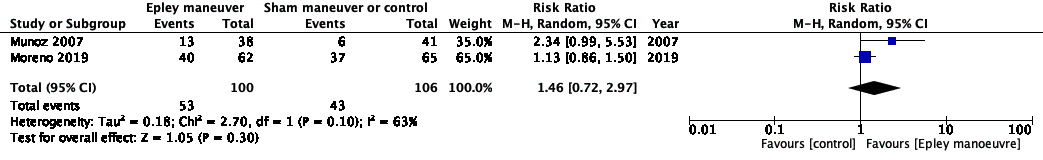


BPPV, benign paroxysmal positional vertigo; DH test, Dix–Hallpike test; CI, confidence interval

Secondary outcomes

1. Disappearance of objective symptoms (nystagmus)


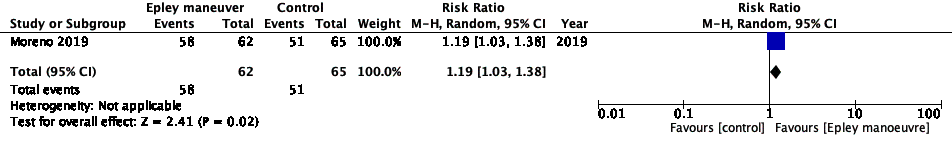


2. DHI-S


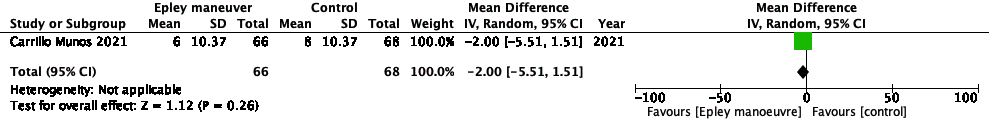


BPPV, benign paroxysmal positional vertigo; DH test, Dix–Hallpike test; CI, confidence interval

**Patients with BPPV in the otolaryngology or subspecialty settings**

Primary outcomes

1. Disappearance of subjective symptoms (vertigo)


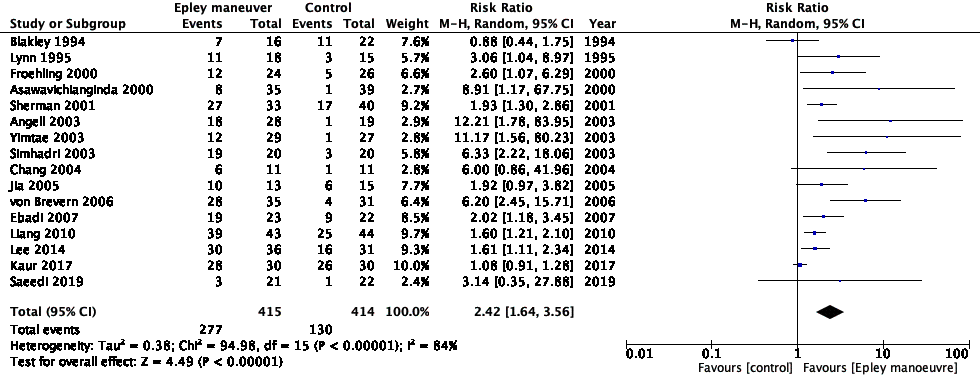


2. Negative findings (DH test)


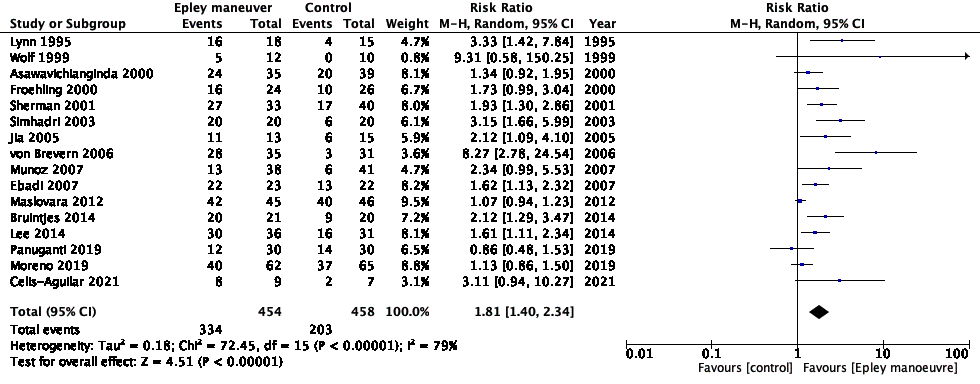


BPPV, benign paroxysmal positional vertigo; DH test, Dix–Hallpike test; CI, confidence interval

Secondary outcomes

1. Disappearance of objective symptoms (nystagmus)


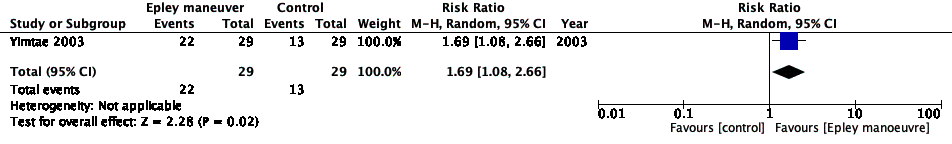


2. DHI-S

**
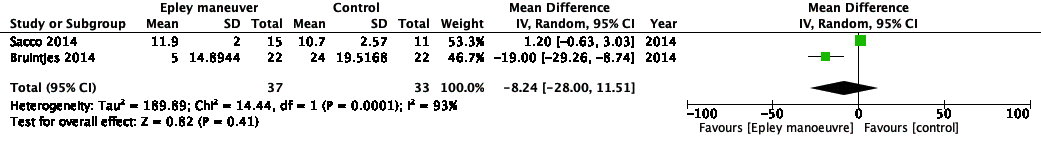
**

DH test, Dix–Hallpike test; CI, confidence interval
