# Supplementary material for: Epley manoeuvre’s efficacy for benign paroxysmal positional vertigo (BPPV) in primary-care and subspecialty settings: a systematic review and meta-analysis
Source: BMC Prim Care. 2023 Dec 2;24:262. doi: 10.1186/s12875-023-02217-z (PMC10693044; doi:10.1186/s12875-023-02217-z)
Supplement: Supplementary file 9 — Additional file 9. Subgroup analysis. [file 12875_2023_2217_MOESM9_ESM.docx]

Additional file 8. Risk of bias table

Patients with BPPV present at primary care clinic

Primary outcomes

1. Disappearance of subjective symptoms (vertigo)

**
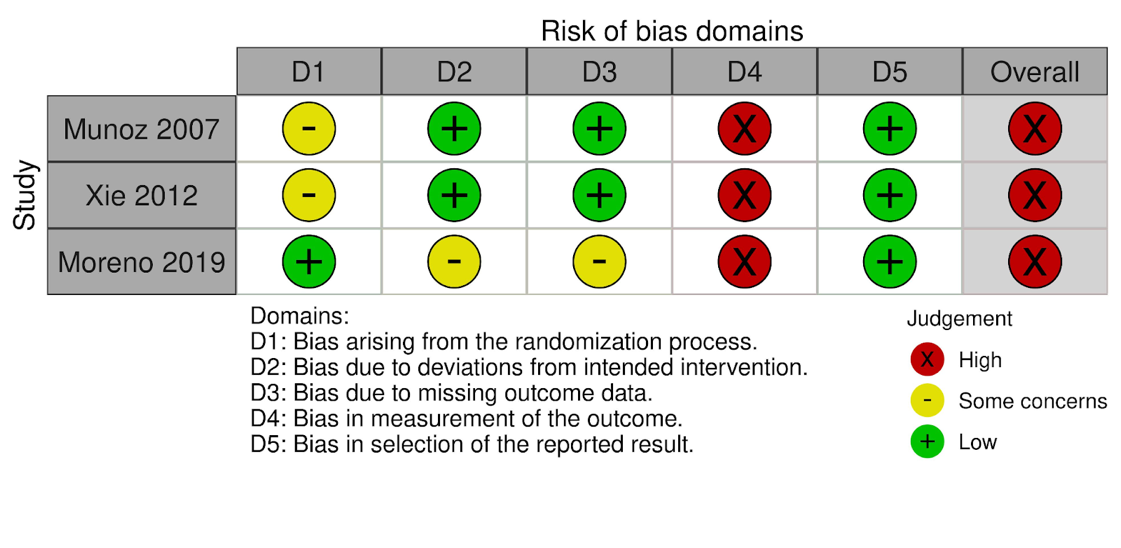
**

**
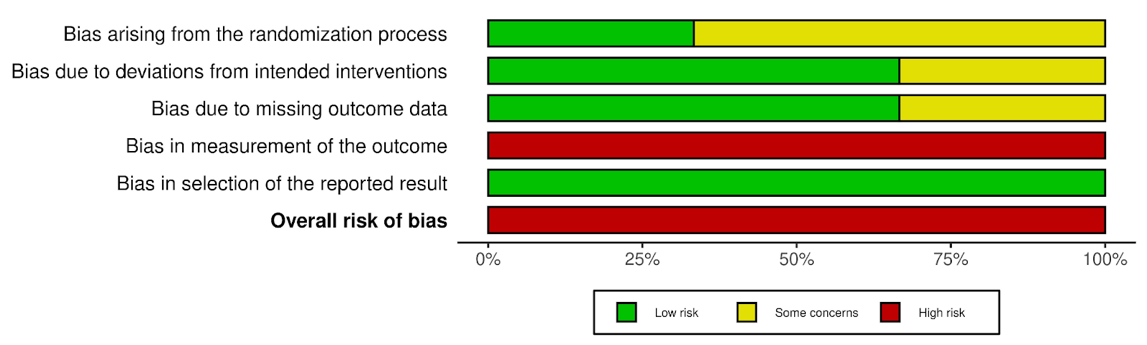
**

BPPV, benign paroxysmal positional vertigo

2. Negative findings (DH test)

**
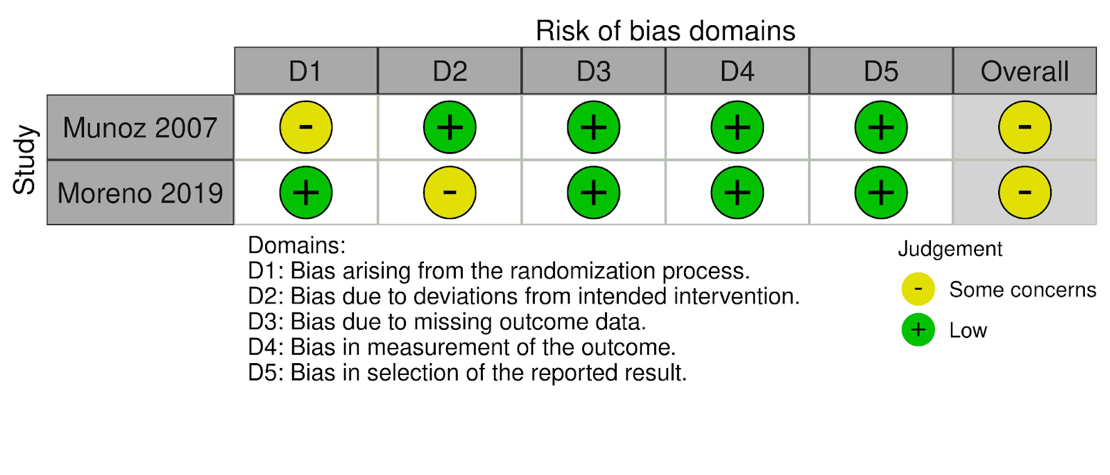
**

**
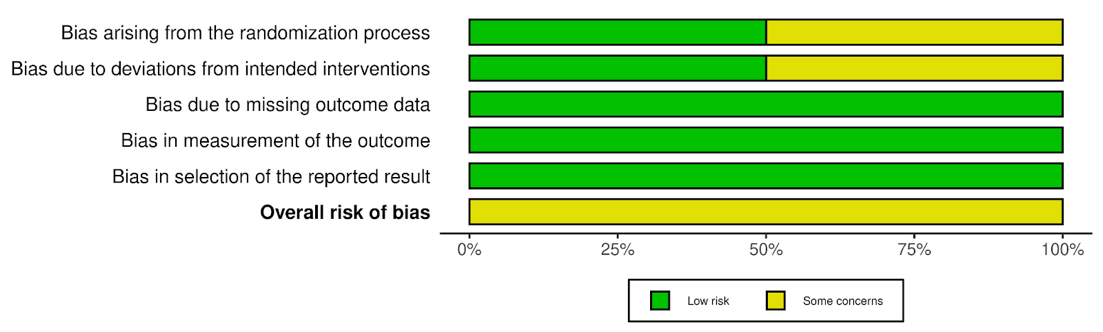
**

DH test, Dix–Hallpike test

3. All adverse event

 None

Secondary outcomes

1. Disappearance of objective symptoms (nystagmus)


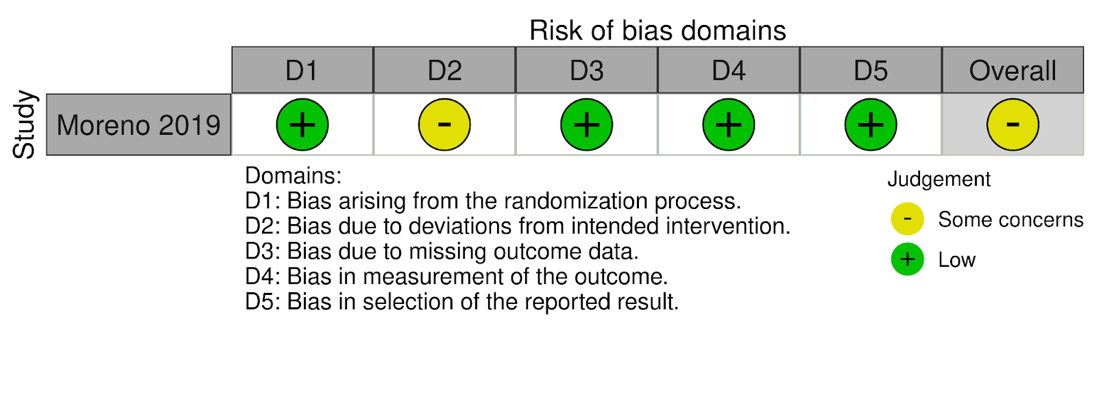


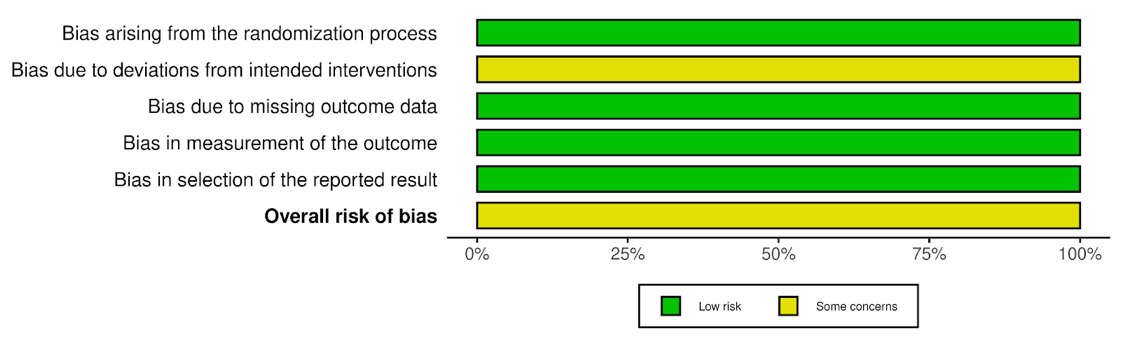


2. DHI-S


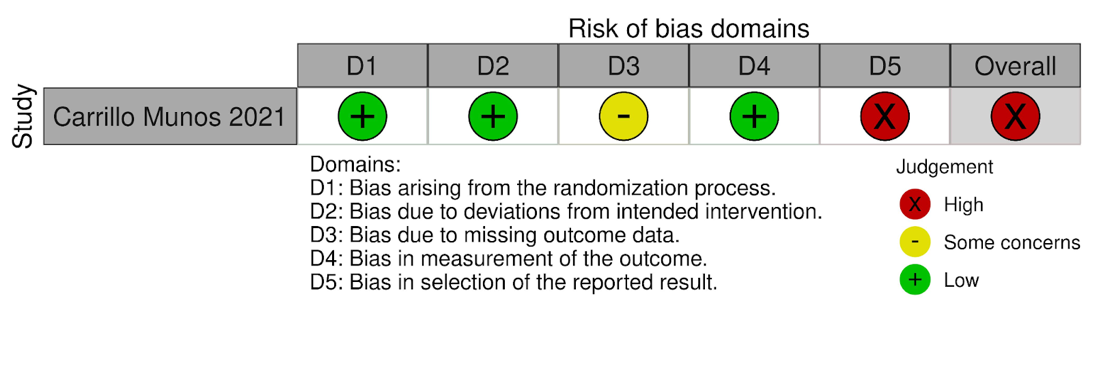


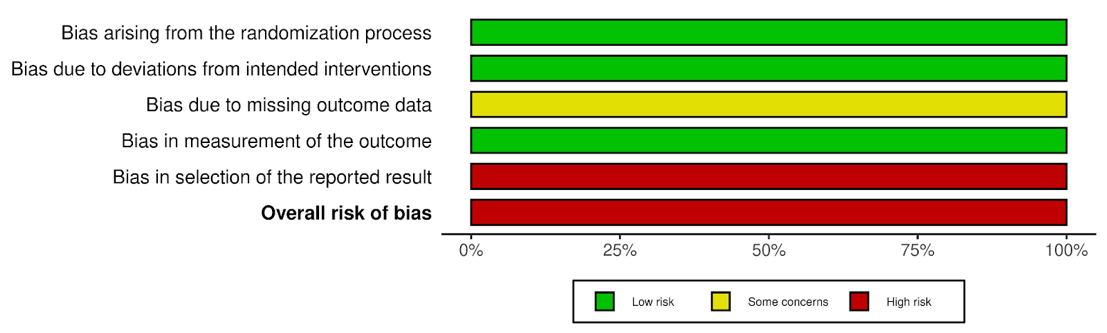


DHI-S, screening version of the Dizziness Handicap Inventory score

Patients with BPPV in otolaryngology or subspecialty settings

Primary outcomes

1. Disappearance of subjective symptoms (vertigo)

**
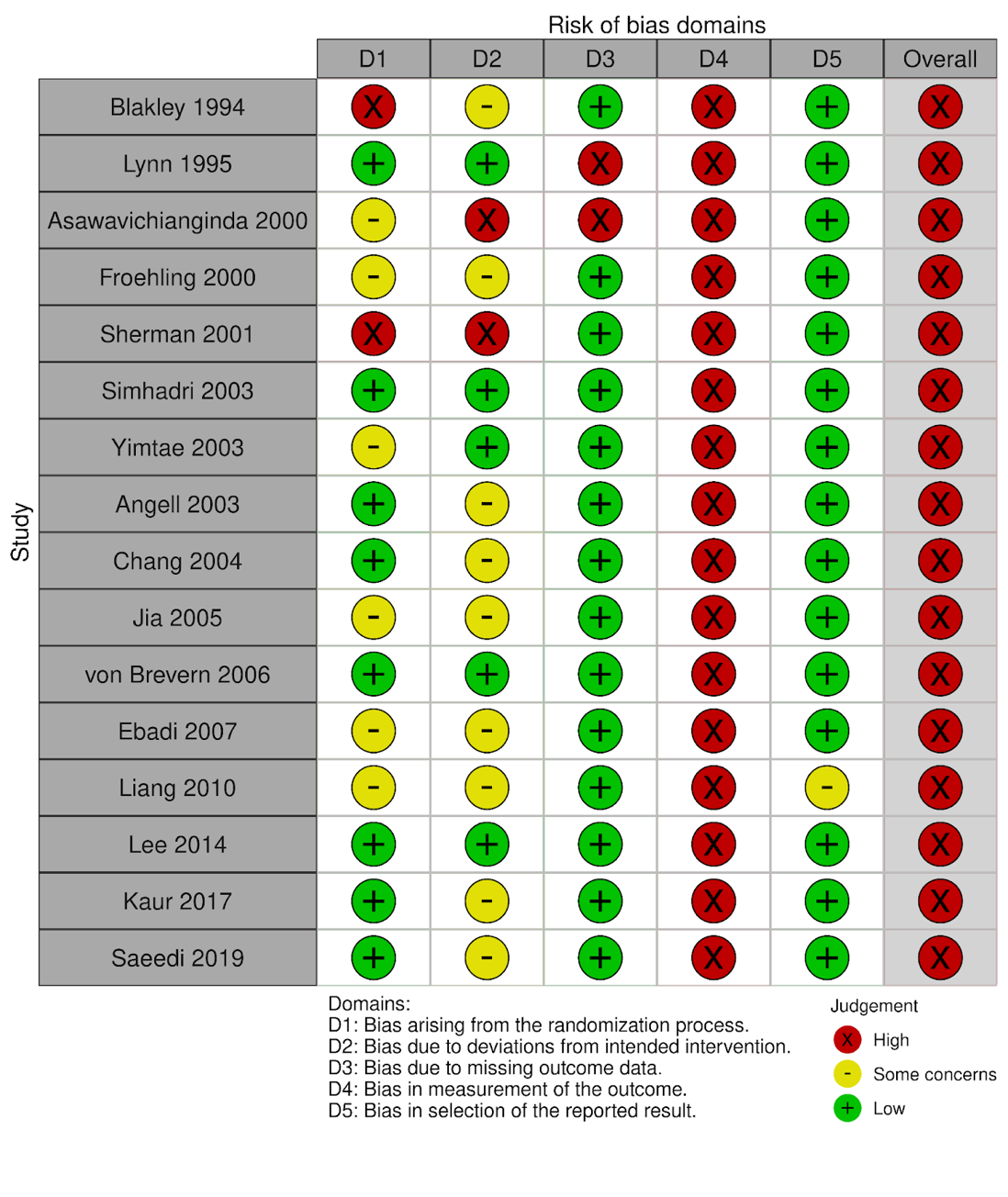
**

**
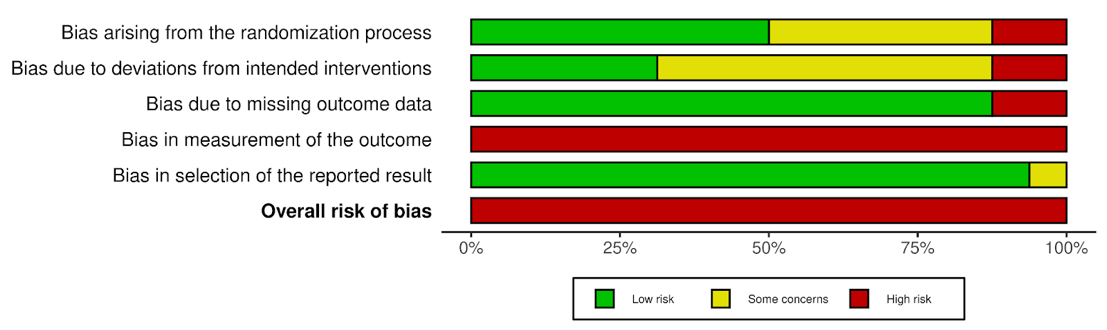
**

BPPV, benign paroxysmal positional vertigo

2. Negative findings (DH test)

**
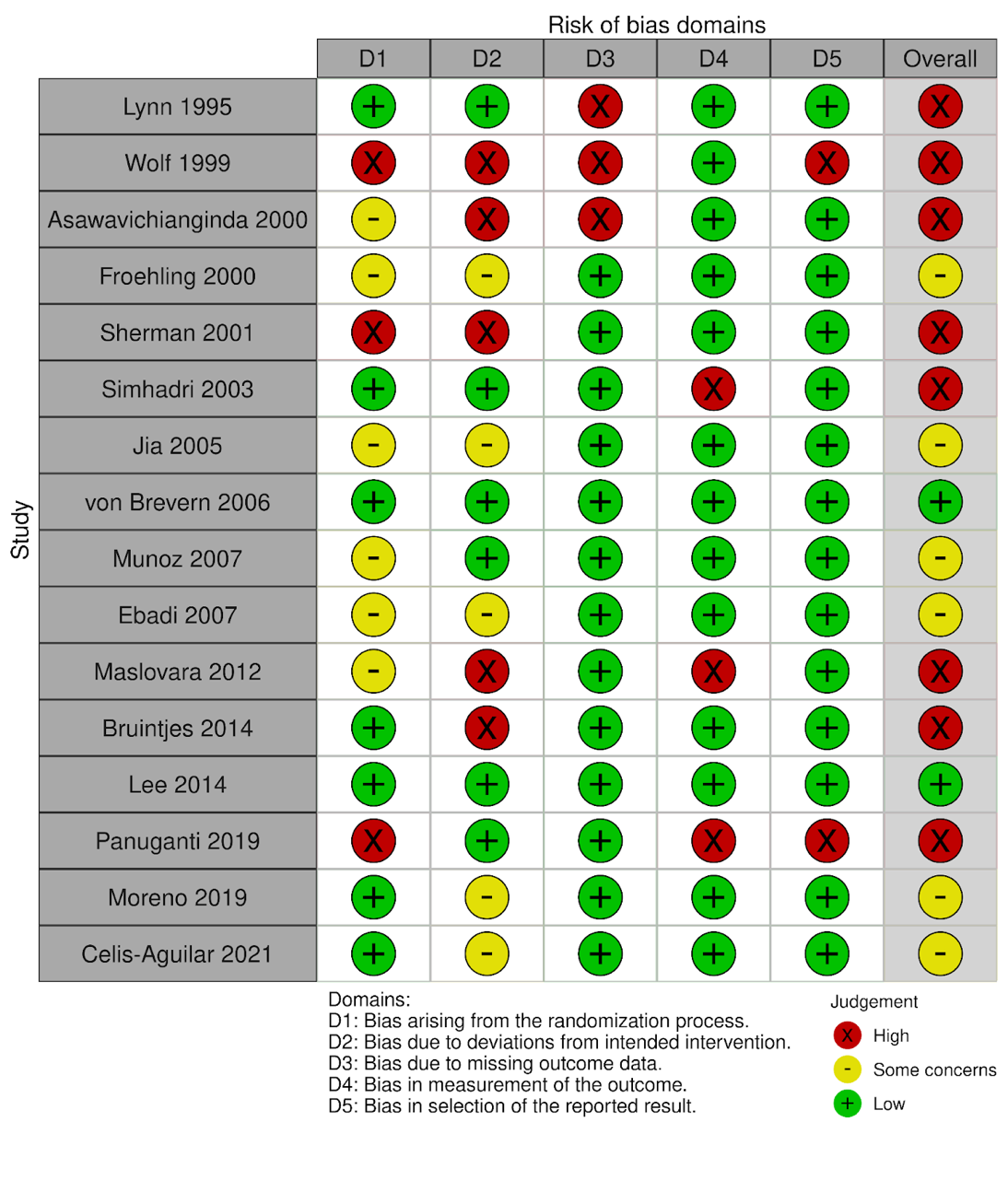
**

**
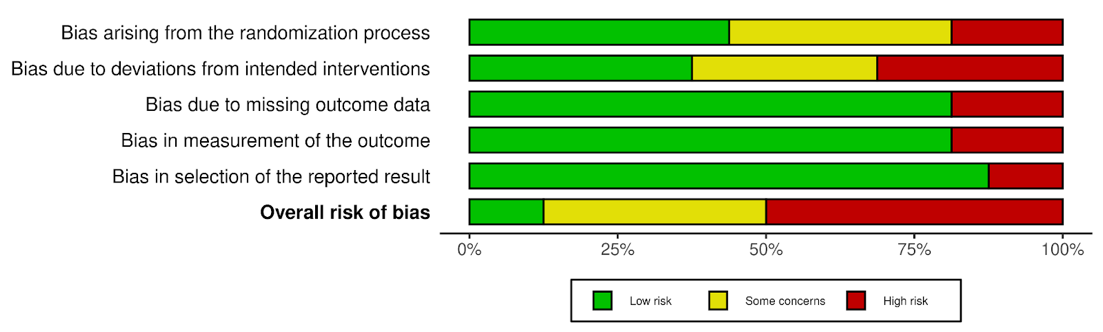
**

DH test, Dix–Hallpike test

3. All adverse event

**
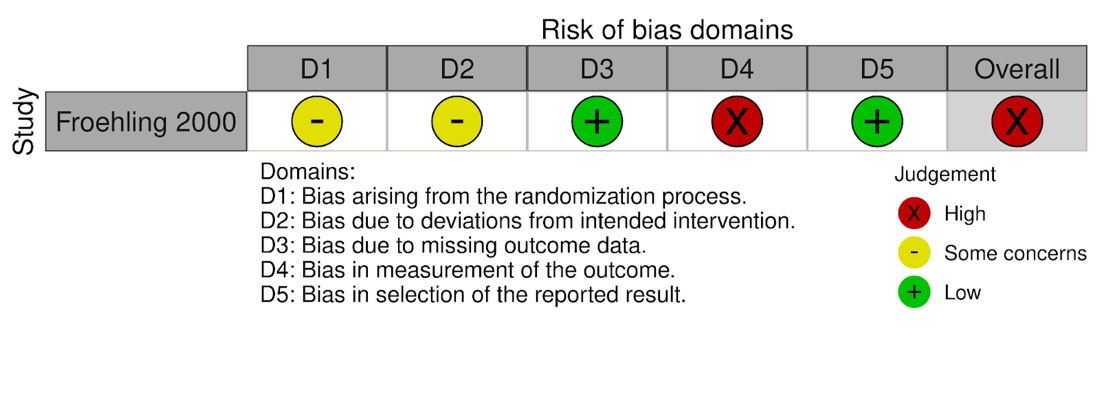
**

**
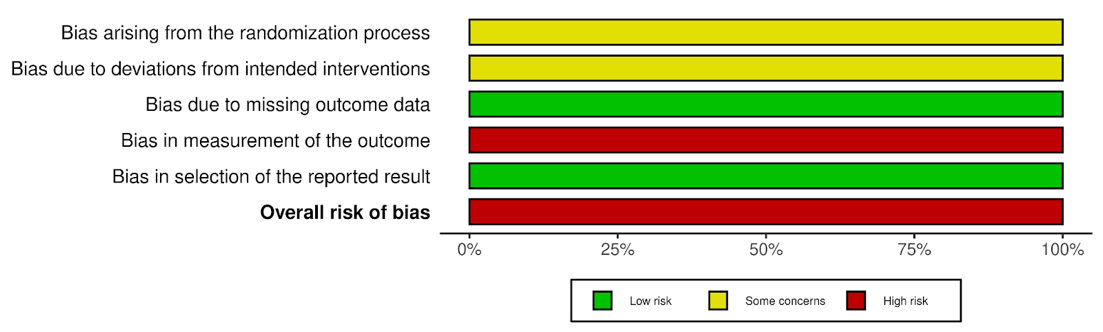
**

Secondary outcomes

1. Disappearance of objective symptoms (nystagmus)


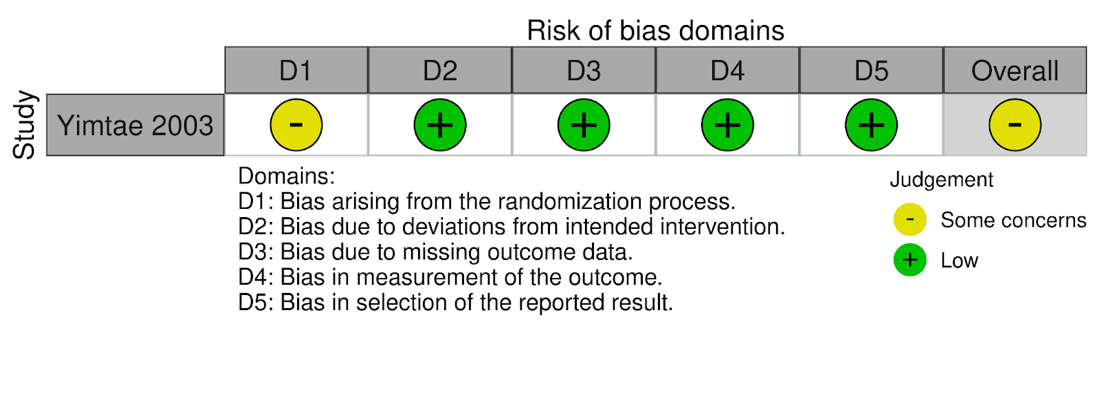


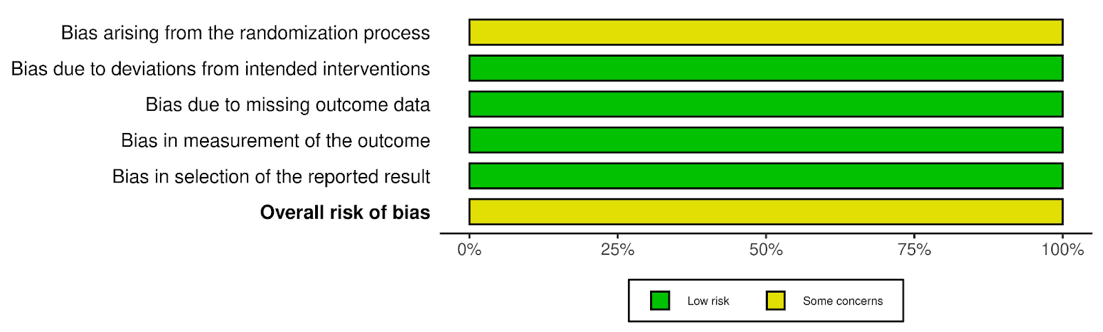


2. DHI-S

**
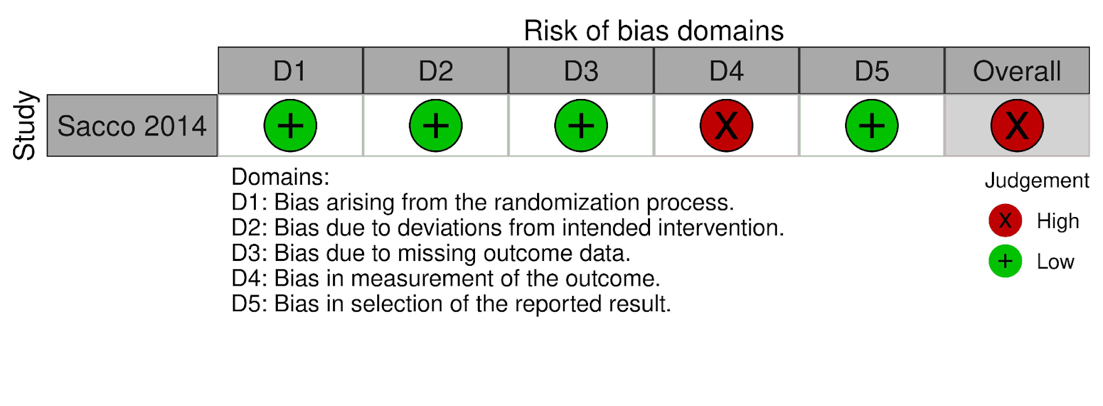
**

**
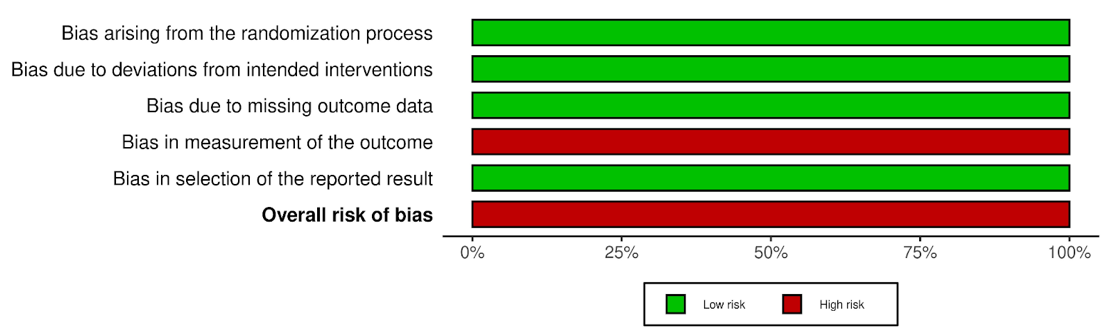
**

DHI-S, screening version of the Dizziness Handicap Inventory score
