# Supplementary material for: Epley manoeuvre’s efficacy for benign paroxysmal positional vertigo (BPPV) in primary-care and subspecialty settings: a systematic review and meta-analysis
Source: BMC Prim Care. 2023 Dec 2;24:262. doi: 10.1186/s12875-023-02217-z (PMC10693044; doi:10.1186/s12875-023-02217-z)
Supplement: Supplementary file 10 — Additional file 10. Sensitivity analysis. [file 12875_2023_2217_MOESM10_ESM.docx]

| Additional file 5. Characteristics of the included studies (otolaryngology or subspecialty settings) (N = 23) | | | | | |  | |
| --- | --- | --- | --- | --- | --- | --- | --- |
| Study Author, Year | Condition/Symptoms, Setting | Comparison | No. Randomised | Outcomes Assessed Here  (Outcome Assessment Tool) | Time of Outcome Assessment | |  |
| Lynn, 1995 | Positive DH test, Otolaryngology | Epley vs Sham manoeuvre | 36 | Disappearance of subjective symptoms (vertigo),  Negative findings (DH test) | 1 week | |  |
| von Brevern, 2006 | A typical history of BPPV and positive DH test, Neurology | Epley vs Sham manoeuver | 67 | Disappearance of subjective symptoms (vertigo),  Negative findings (DH test) | 1 day | |  |
| Yimtae, 2003 | Positive DH test, Otolaryngology | Epley plus Drug vs Drug | 58 | Disappearance of subjective symptoms (vertigo),  Disappearance of objective symptoms (nystagmus) | 1 week | |  |
| Lee, 2014 | A typical history of BPPV and positive DH test, Otolaryngology | Epley vs Sham manoeuver | 68 | Disappearance of subjective symptoms (vertigo),  Negative findings (DH test) | First visit | |  |
| Kaur, 2017 | A typical history of BPPV and positive DH test, Otolaryngology | Epley or Epley plus Betahistine vs Drug | 60 | Disappearance of subjective symptoms (vertigo) | 1 week | |  |
| Cohen, 2005 | Positive DH test, Otolaryngology | Epley vs Sham manoeuver | 49 | Vertigo frequency and Vertigo intensity | 1 week | |  |
| Sherman, 2001 | A typical history of BPPV and positive DH test, Otolaryngology | Epley vs Sham manoeuver or no treatment | 86 | Disappearance of subjective symptoms (vertigo),  Negative findings (DH test) | 2 weeks | |  |
| Wolf, 1999 | A typical history of BPPV and positive DH test, Otolaryngology | Epley vs no treatment | 22 | Negative findings (DH test) | First visit | |  |
| Sridhar, 2003 | A typical history of BPPV and positive DH test, Otolaryngology | Epley vs Sham manoeuver | 40 | Disappearance of subjective symptoms (vertigo),  Negative findings (DH test) | 1 week | |  |
| Saeedi, 2019 | A typical history of BPPV and positive DH test, Otolaryngology | Epley vs Drug | 44 | Negative findings (DH test) | second week after intervention | |  |
| Sacco, 2014 | Positive DH test, Emergency room | Epley plus rehabilitation vs Drug | 26 | Dizziness Handicap Inventory | 1 month | |  |
| Panuganti, 2019 | A typical history of BPPV and positive DH test, Otolaryngology | Epley vs Drug | 60 | Negative findings (DH test) | First visit | |  |
| Maslovara, 2012 | Positive DH test, Otolaryngology | Epley vs Drug | 96 | Negative findings (DH test) | 1 week | |  |
| Liang, 2010 | A typical history of BPPV and positive DH test, Otolaryngology | Epley plus Drug vs Drug | 87 | Disappearance of subjective symptoms (vertigo) | 4 days | |  |
| Jia, 2005 | A typical history of BPPV and positive DH test, Emergency room | Epley vs Drug | 28 | Disappearance of subjective symptoms (vertigo),  Negative findings (DH test) | 1 day | |  |
| Froehling, 2000 | A typical history of BPPV and positive DH test, Emergency room | Epley vs Sham manoeuver | 50 | Disappearance of subjective symptoms (vertigo),  Negative findings (DH test),  All adverse events | 1 week | |  |
| Ebadi, 2007 | A typical history of BPPV and positive DH test, Neurology and Otolaryngology | Epley vs Drug | 45 | Disappearance of subjective symptoms (vertigo),  Negative findings (DH test) | 1 week | |  |
| Chang, 2004 | A typical history of BPPV and positive DH test, Emergency room | Epley vs Sham manoeuver | 22 | Disappearance of subjective symptoms (vertigo) | First visit | |  |
| Bruintjes, 2014 | A typical history of BPPV and positive DH test, Otolaryngology | Epley vs Sham manoeuver | 44 | Negative findings (DH test),  Dizziness Handicap Inventory | 1 month | |  |
| Blakley, 1994 | A typical history of BPPV and positive DH test, Otolaryngology | Epley vs no treatment | 38 | Disappearance of subjective symptoms (vertigo) | 1 month | |  |
| Asawavichianginda, 2000 | A typical history of BPPV and positive DH test, Neurology | Epley vs no treatment | 85 | Disappearance of subjective symptoms (vertigo),  Negative findings (DH test) | 1 week | |  |
| Angell, 2003 | A typical history of BPPV and positive DH test, Neurology | Epley vs no treatment | 47 | Disappearance of subjective symptoms (vertigo) | 1 month | |  |
| Celis-Aguilar, 2021 | A typical history of BPPV and positive DH test, Otolaryngology | Epley vs Sham manoeuver | 19 | Negative findings (DH test),  Dizziness Handicap Inventory | 1 week | |  |

DH test, Dix–Hallpike test; BPPV, benign paroxysmal positional vertigo
